# The autistic-like behaviors development during weaning and sexual maturation in VPA-induced autistic-like rats is accompanied by gut microbiota dysbiosis

**DOI:** 10.7717/peerj.11103

**Published:** 2021-05-03

**Authors:** Qingmin Kong, Peijun Tian, Jianxin Zhao, Hao Zhang, Gang Wang, Wei Chen

**Affiliations:** 1 State Key Laboratory of Food Science and Technology, Jiangnan University, Wuxi, P. R. China; 2 School of Food Science and Technology, Jiangnan University, Wuxi, China; 3 International Joint Research Laboratory for Probiotics & Gut Health, Jiangnan University, Wuxi, P. R. China; 4 (Yangzhou) Institute of Food Biotechnology, Jiangnan University, Wuxi, P. R. China; 5 National Engineering Center of Functional Food, Jiangnan university, Wuxi, China; 6 Wuxi Translational Medicine Research Center and Jiangsu Translational Medicine Research Institute Wuxi Branch, Wuxi, China; 7 Beijing Innovation Centre of Food Nutrition and Human Health, Beijing Technology and Business University, Beijing, China

**Keywords:** Autism spectrum disorder, Weaning, Sexual maturation, Behavioristics, Gutmicrobiota

## Abstract

Researches on gut microbiota in autism have mostly focused on children, but the dynamic changes of gut microbiota from weaning to adulthood were still not clear because of the difficulty of diagnosing autism. In this study, autistic-like rats indued by valproate (VPA) were tracked from weaning (end of breastfeeding; four weeks old) to sexual maturation (food; eight weeks old). Autistic-like rats were found to show obvious developmental disorders. During weaning, autistic-like rats only exhibited obvious repetitive stereotyped behaviors, but the autistic-like behaviors were fully apparent upon sexual maturation. Significant differences were observed between the gut microbiota of autistic-like and healthy rats across both age groups. The correlation analysis results revealed that the correlation between behaviors and some microbiota, especially *Helicobacter*, did not vary with age or diet. The total amount of short-chain fatty acids (SCFAs) decreased, butyric acid metabolism decreased, and propionic acid metabolism increased in the feces of autistic-like rats. The correlation between autistic-like behaviors and the butyric acid and propionic acid levels did not vary with diet or age. Inositol phosphate metabolism, amino acid metabolism, and lipopolysaccharide biosynthesis were significantly associated with autistic-like behaviors. Our results showed that although the microbiota and SCFAs related to autism were affected by age and diet, some remained consistent irrespective of age and diet, and they could be considered two of the factors related to autistic-like behaviors development.

## Introduction

Autism spectrum disorder (ASD) has been recognized as a broad spectrum of neurodevelopmental disorders frequently occurring in infants and young children. It is characterized by impaired social communication (both verbal and non-verbal) and repetitive stereotyped behavior, often accompanied by intellectual disability, epilepsy, or hyperactivity, as well as other clinical symptoms. ASD onset usually occurs before four years of age (Lord et al., 2018). According to the latest data released by the Centers for Disease Control and Prevention, the prevalence of ASD was 1:59 in 2018 (an increase of 15% from 1:68 in 2016), with the prevalence in boys being four to five times higher than that in girls (Oien, Cicchetti & Nordahl-Hansen, 2018).

Therefore, genetic factors alone cannot fully explain the rapidly increasing incidence of ASD. As such, increasing attention is being paid to the role of non-genetic factors in the pathogenesis of ASD, such as abnormalities in immune regulation, neurotransmitters or signal transmission, and environmental risk factors (Masi et al., 2017).

Prenatal or perinatal drugs, viral infections, and heavy metal poisoning can increase the risk of ASD in offspring. Valproic acid is used to treat various types of seizure disorders in humans. Population studies have shown that the offspring of mothers who take VPA during pregnancy are seven times more likely to develop ASD (Evatt et al., 2009). Also, Liu et al. (2018) provide evidence that offspring whose mothers were intraperitoneally injected with sodium VPA on an embryonic day 12.5 could mimic the microbiome features of autism, whereas as far back as 2005 (Schneider & Przewłocki, 2005), the model has been described as having behavioral alterations similar to autism in humans. Further, severe viral infections in the first trimester or severe bacterial infections in the second trimester increase the risk of autism in the offspring by four times and 1.42 times, respectively (Zhao et al., 2019).

Clinical studies have shown that the gut microbiota plays a vital role in the two-way communication between the gastrointestinal tract and the central nervous system (CNS) and are especially closely related to the development of CNS dysfunctions and disorders. Gastrointestinal dysfunction occurs in 40% of patients with ASD. As a highly heterogeneous neurodevelopmental disorder, ASD is accompanied by diverse degrees of intestinal dysfunction, indicating high heterogeneity in the gut microflora composition. Studies have shown that the abundance of the Firmicutes phylum is lower, that of the Bacteroidetes phylum is higher, and the Bacteroidetes/Firmicutes ratio is higher in the feces of autistic children compared with healthy children. The abundances of the *Streptococcus*, *Bifidobacterium*, *Prevotella*, *Coprococcus,* and *Veillonella* genera in autistic patients are also atypical (Hughes, Rose & Ashwood, 2018). Also, duodenal biopsies of autistic patients with gastrointestinal disorders have shown a higher abundance of the *Sutterella* genus, which is associated with mucosal metabolism. The disruption of the mucosal barrier due to an imbalance of the gut microflora may lead to increased intestinal permeability and pro-inflammatory cytokines (Kushak et al., 2011). Notably, to date, studies on the gut microbiota of autistic patients have mostly focused on children, and few studies have investigated the dynamic changes in the gut microbiota from infancy to adulthood because of the difficulty of diagnosing autism in infants. Thus, in this study, the behaviors, gut microbiota, and short-chain fatty acid (SCFA) metabolism of VPA-treated rats were tracked from weaning to sexual maturation to investigate the relationship between the gut microbiota, SCFAs, and autistic-like behaviors development.

## Materials & Methods

### Animal model

Wistar rats (initial body weight: males, 280–290 g, *n* = 12; females, 220–250 g, *n* = 6) were purchased from Beijing Vital River Laboratory Animal Technology Co., Ltd. for use in our experiments. The rats were housed in the experimental animal center of Jiangnan University under standard conditions at a constant room temperature of 25 °C ± 2 °C, constant humidity of 50% ± 5%, and a 12 h light-dark cycle. After acclimating for 1 week, the rats were caged at 17:00 with a male: female ratio of 2:1. The day on which sperm was observed in vaginal smears was designated as embryonic day 1 (E 1). The pregnant rats were then kept in a separate cage and divided into two groups: the experimental group (*n* = 6) and the control group (*n* = 6). At E 12.5, the pregnant rats in the experimental group (*n* = 6) were intraperitoneally injected with 600 mg/kg of sodium VPA (sodium VPA powder was mixed with normal saline (NS) to obtain a 250 mg/ml solution) (Tian et al., 2014), whereas those in the control group (*n* = 6) were injected with NS (Matsuo, Yabuki & Fukunaga, 2017). Schneider et al. found that, compared with the behavioral performance of VPA-induced autistic female rat, the behavior of VPA-induced autistic male rat was more similar to the clinical performance of autistic patients; therefore, in this study, we selected male offspring as the experimental animals (Schneider et al., 2008). On postnatal day 21, the male offspring of the mother rats injected with normal saline were randomly divided into a control group (*n* = 6) and the male offspring of the mother exposed to VPA were randomly divided into an autism group (*n* = 6). In pre-experiment, we found that tooth deformity occurred in a part of VPA-induced autistic rats, which is gradually severe after eating independently (age of 4 weeks). Thus, the rats with tooth deformities must be kicked out to avoid the great effect on the living quality of autistic rats. For these considerations, to ensure the parallelism of the experiment, 6 rats with healthy teeth were finally chosen in every group for the experiment. This study was approved by the Ethics Committee of Experimental Animals in Jiangnan University, China (JN. No. 20180915S0601230[183]), and the procedures were conducted under the European Community Guidelines for the Care and Use of Experimental Animals (Directive 98 2010/63/EU).

### Growth and development

#### Body-weight monitoring

The weight of the offspring rats in the autistic group and healthy group were checked on the 7th, 21st, and 56th days after birth.

#### Incline test

On the 7th, 8th, 9th, and 10th days after birth, the time taken by rats in the two groups to rotate 180° on a 25° smooth slope in a downward-facing direction was recorded (Zhou et al., 2018). Because the seven-day-old rats took a long time to rotate, a scoring principle was adopted as follows: a rotation angle of 0°–45° was awarded 0.25 points, of 90° 0.5 points, of 90°–180° 0.75 points, and 180° 1 point.

#### Swimming coordination

Swimming ability was recorded at the ages of 7, 11, and 13 days. The temperature of the water was 25 °C ± 2 °C, and the swimming time was 10 s. After swimming, the rats were dried with sterile towels and put back into the cage. The following scoring criteria were adopted: head-on water and nose underwater = 1 point; head and nose above or equal to the water surface with ears underwater = 2 points; head and nose on or equal to the water surface with the water level around the middle of the ears = 3 points; ears above the water level = 4 points (Zhou et al., 2018).

### Behavior test

#### Open-field test

The core features of autism are frequently accompanied by other manifestations, including limited environmental exploration. The open-field test is a straight-forward test to investigate the activity, locomotor activity, and exploratory behavior in rodents. An open field reaction box of 100 × 100 × 60 cm (length × width × height) in size and with blackened inner walls was used for this test. Locomotor activity was monitored using a video tracking system (EthoVision pro, Noldus Inc., Leesburg, VA). EthoVision software was used to calculate the time spent in the center. The field of vision of the camera covered the whole interior of the open field box. The test time for each rat was 6 min. After testing each rat, 75% (v/v) ethanol was used to wipe the box (Simmons et al., 2020).

#### Repetitive or stereotyped behavior test

Repetitive or stereotyped behavior is described as a symptom of autism in the DSM-5. In the animal model, repetitive self-grooming is a measure of the repetitive behavior characteristic of autism. As such, this behavior was chosen as a reflection of repetitive or stereotyped behavior (Ranjana & Anurag, 2015). The rats were placed in an empty cage with no padding for 6 min and then placed in another cage, where the number of times they groomed was recorded for 6 min. After testing each rat, 75% (v/v) ethanol was used to wipe the box (Simmons et al., 2020).

#### Social test

On the 35th day after birth, six rats from each group with a birth time difference of no more than 1 day and a body mass difference of less than 15 g were selected for the social test. The test was performed in a three-chamber cage of 100 × 100 × 60 cm (length × width × height) in size. The experimental device was composed of three rectangular boxes, each with dimensions of 100 × 30 × 60 cm (length × width × height). The partition between each box was transparent plexiform glass, and there was a channel in the middle to connect the three boxes. One day before the experiment, all selected rats were placed in the testing room to allow them to adapt to the environment. The following day, during the experiment, a test rat was placed in the middle chamber to adapt for 10 min. An empty wire cage was then placed in the left compartment, and a strange rat was placed in the right compartment and covered with a wire cage. The channels leading from the middle chamber to the left and right chambers were opened, and the behavior of the test rat was recorded with a video camera for 10 min. The social index was the ratio of the time spent in the right chamber (with the strange rat) to the time spent in the left chamber (Simmons et al., 2020).

#### Novel object recognition test

The rat novel object recognition test, first proposed by Ennaceur & Delacour (1988), consists of three phases: the adaptation phase, the familiarity phase, and the test phase.

(1) During the adaptation phase, the rats were placed successively in the experimental box without any object so that they could explore freely to adapt to the experimental environment and minimize stress.
(2) During the familiarity phase, two identical objects (A1 and A2) were placed in the adjacent or opposite positions of the bottom plate of the experimental box, and the rats were placed in the experimental box with their backs to the two objects. After 10 min, the rats were removed and placed back in the animal cage. After a 10 min interval, the test phase was started.
(3) The procedure of the test phase was similar to that of the familiarity phase, except that one of the two identical objects was replaced by a novel object. Thus, the objects are called the familiar object (A3) and the novel object (B). In the test phase, to prevent a situation wherein the experimental rat has a special preference for a certain object or a certain position, which may introduce experimental errors, the functions and positions of the familiar and novel objects were changed during each experiment for each successive rat. The time spent on the side of the novel object was used to assess cognitive ability.

### Gut microbiota analysis

DNA from the feces samples of autistic-like rats (*n* = 6) and healthy rats (*n* = 6) collected on the 21st and 56th days after birth was obtained using a FastDNA® Spin Kit for Stool (MP Biomedicals, Santa Ana, CA, USA), following the manufacturer’s instructions. The V3-V4 region of the 16S rRNA was amplified with universal primers (341F and 806R), and products were purified with aTIANgel Mini Purification Kit (TIANGEN, Beijing, China) and quantified using a Qubit dsDNA HS Assay Kit (Life Technologies Corporation, Carlsbad, CA, USA). Pair-end reads with an overlap of >10 bp and without mismatches were assembled. The operational taxonomic unit (OTU) was established de novo using uclust with 97%. The first sequence in each OTU cluster was chosen as the representative sequence and then aligned to the SLIVA core set in QIIME using the PyNAST aligner (Mao et al., 2018).

### Short-chain fatty acid analysis

Feces (50–100 mg) was freeze-dried and soaked in 500 µL of saturated sodium chloride solution for 30 min. After homogenization, 40 µL of 10% sulfate was added. After homogenization, 1 ml of ethyl ether homogenate was added, and the mixture was centrifuged at 12,000 g for 15 min at 4 °C. Then the 500 µL supernatant was filtered through a 0.22 µm filter membrane and transferred to a gas bottle for GC-MS analysis (Wang et al., 2017). Concentrations were calculated using reference acid (acetic acid, propionic acid, butyric acid, and valeric acid purchased from Sinopharm Chemical Reagent Co., Ltd.; isobutyric acid and isovaleric acid purchased from Sigma).

### Statistical and analytical methods

Statistical analyses were performed using SPSS 20.0 and GraphPad Prism 7. The data were assessed for normal distribution presented as mean ± standard deviation (SD) and plotted as means with 95% confidence intervals. The significant difference was evaluated using one-way analysis of variance (ANOVA) and Fisher’s least significant difference (LSD) tests. Alpha (*α*) diversity (Chao1, ACE, Shannon, Simpson, and Fisher indexes) and beta (*β*) diversity were assessed by principal coordinate analysis (PCoA) performed using R software. Linear discriminant analysis Effect Size (LEfSe) was performed online (http://huttenhower.sph.harvard.edu/galaxy). Linear discriminant analysis (LDA) Effect Size was used to differentiate between biomarkers (Wilcoxon rank-sum test, *α* <0.01, log LDA >2) (Segata et al., 2011; Zhai et al., 2019). Differential pathways based on the Kyoto Encyclopedia of Genes and Genomes (KEGG) orthology were identified using Phylogenetic Investigation of Communities by Reconstruction of Unobserved States (PICRUST) and presented using STAMP (Langille et al., 2013). The correlation analyses using Pearson correlation for pairwise complete data were performed in SPSS 20.0, and the heatmap figures were presented by R 3.6.1.

## Results

### VPA-treated rats presented early (4 weeks of age or before) developmental abnormalities

**Figure 1:**
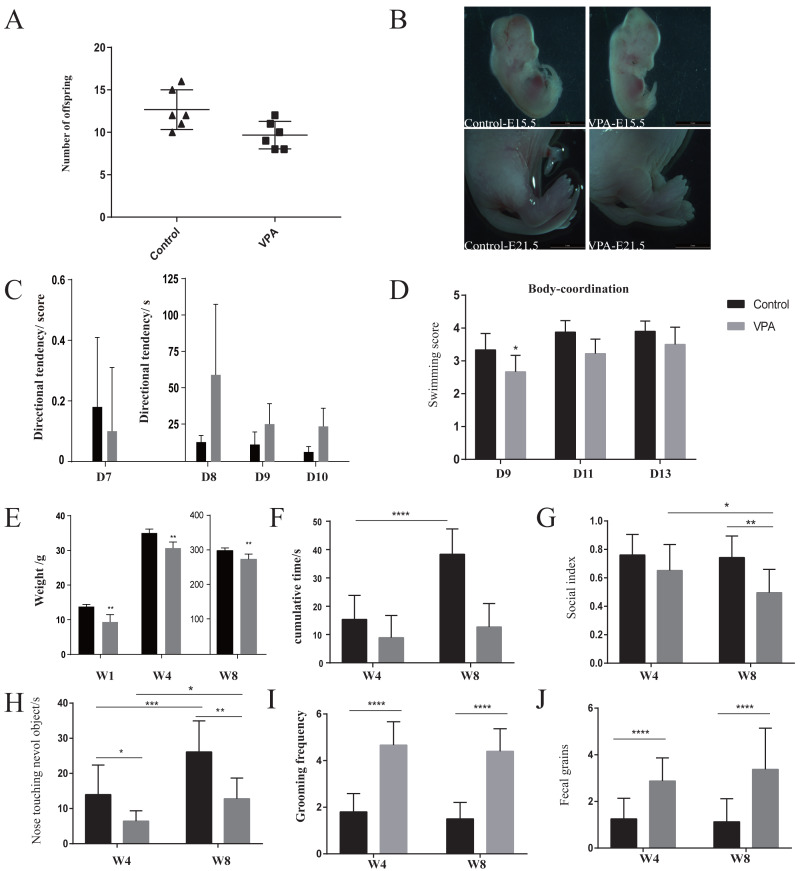
Development and behaviours. (A) Fertility rate in the autism and healthy groups. (B) Observation of rat tail deformity with an inverted fluorescence microscope. (C) Swimming coordination. (D) Incline test. (E) Body weight monitoring. (F) Open-field test: time spent in the middle area of an open field. (G) Social test: the index of social skill. (H) Novel object recognition: time spent in staying around a novel object. (I) Repetitive or stereotyped behaviour: repetitive self-grooming. (J) The number of times autistic rats defecated: anxiety-like behaviour. Data represent mean with 95% CI. One-way ANOVA followed by Fisher’s LSD multiple-comparison test: *(*p* < 0.05); **(*p* < 0.01); ***(*p* < 0.001); ****(*p* < 0.0001).

The number of offspring in VPA-treated group was significantly lower than that of healthy group (Fig. 1A). VPA-treated rats presented deformed tails, which indicating that VPA exposure occurred in utero (Fig. 1B). Direction tendency (Fig. 1C) and swimming coordination (Fig. 1D) were also assessed to evaluate development. Also, a slower increase of weight showed in VPA-treated group (Fig. 1E). Direction tendency and swimming scores showed that autistic rats had weak early direction ability, poor swimming coordination, and obvious abnormal development.

### Autistic-like behaviors gradually appeared in VPA-treated rats from weaning to sexual maturity

Behavioral monitoring using the open field test from weaning until sexual maturity granted insights into loco-motor activity and exploratory behavior. The decreased entries and time spent in the center of the arena are indicative of anxiety-like behavior in VPA-treated group compared with healthy group (Fig. 1F). With age, the exploratory behavior of healthy group increased, but that of the VPA-treated rats did not improve significantly. The times of VPA-treated rats defecated increased significantly during exploration behavior (Figs. 1G and 1J), indicating that they were anxious in the unfamiliar environment. The cognitive ability of all immature rats in both groups was weak early on, but in the novel object recognition experiment (Figs. 1F and 1H), the VPA-treated rats showed significant cognitive impairment compared with healthy rats. At eight weeks of age, although the cognitive ability of had improved VPA-treated rats, it was still significantly impaired compared with healthy rats. In the three-chamber cage social experiment (Fig. 1G), four-week-old VPA-treated rats began showing weak social skills. After sexual maturity, their social index was significantly lower than healthy rats, and they showed obvious signs of social impairment. Further, the frequency of self-grooming, one of the main indicators of repetitive stereotyped behavior, was longer among the four- to eight-week-old autistic rats than among the healthy rats, and the frequency of self-grooming among VPA-treated rats remained stable during this period, indicating repetitive or stereotyped behavior (Fig. 1I). Thus, in this VPA-induced model, rats presented autistic-like behaviors. The repetitive or stereotyped behavior first significantly appeared by four weeks. Other behaviors, especially social skills, were also abnormal but not significant. We did not monitor more time point from weaning to sexual maturation identifying the time of significant anomalies, so it needs more point in time monitoring for further confirmation.

### Autistic-like rats showed gut microbiota imbalance from weaning to sexual maturation

**Figure 2:**
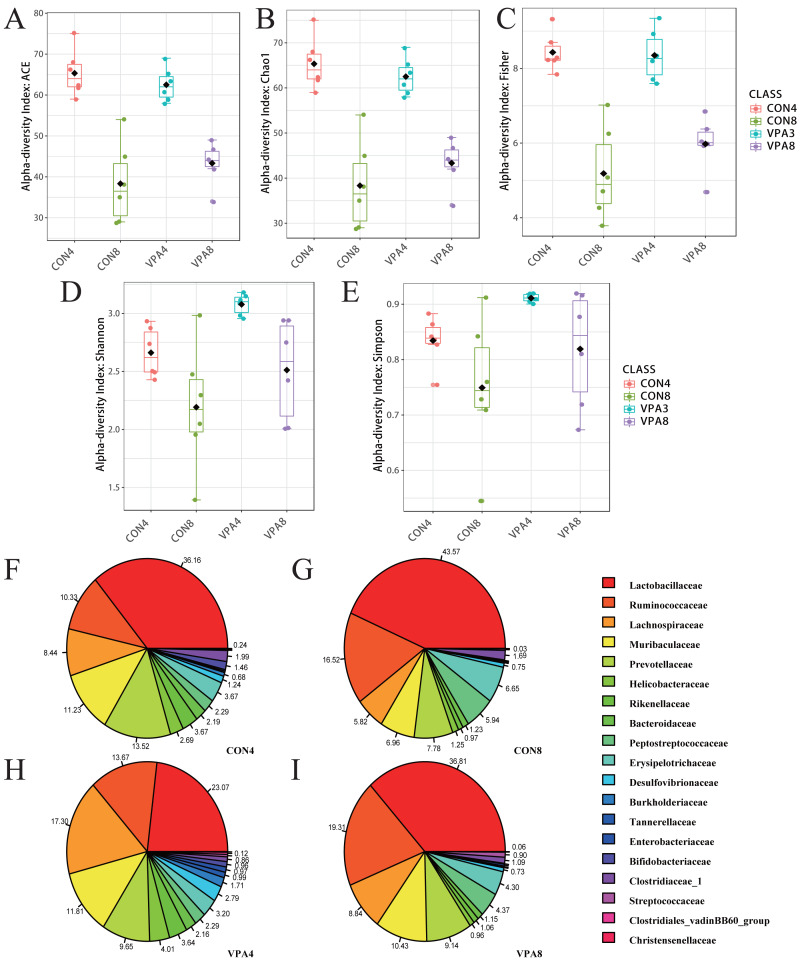
Microbiota analysis. (A) *α*-diversity evaluated by Chao1. (B) *α*-diversity evaluated by ACE. (C) *α*-diversity evaluated by Fisher. (D) *α*-diversity evaluated by Shannon. (E) *α*-diversity evaluated by Simpson. (F) *α*-diversity evaluated by Fisher indexes. (G-I) Pie chart of the abundances of major families. Data represent mean with 95% CI. One-way ANOVA followed by Fisher’s LSD multiple-comparison test: *(*p* < 0.05); **(*p* < 0.01).

The intestinal microflora composition of the healthy and autistic-like rats varied with development from four to eight weeks of age. As shown in Fig. 2A, the ACE and Chao1 indexes exhibited a declining trend with age in both the healthy and autistic-like rats, suggesting that the abundance of total intestinal microbiota decreased in both groups. Besides, the Shannon, Simpson, and Fisher indexes also showed a declining trend with age in both groups. Despite the nonsignificant difference in the abundance of intestinal microbiota between the two groups, the diversity of intestinal microbiota was higher in autistic-like rats than healthy rats.

**Figure 3:**
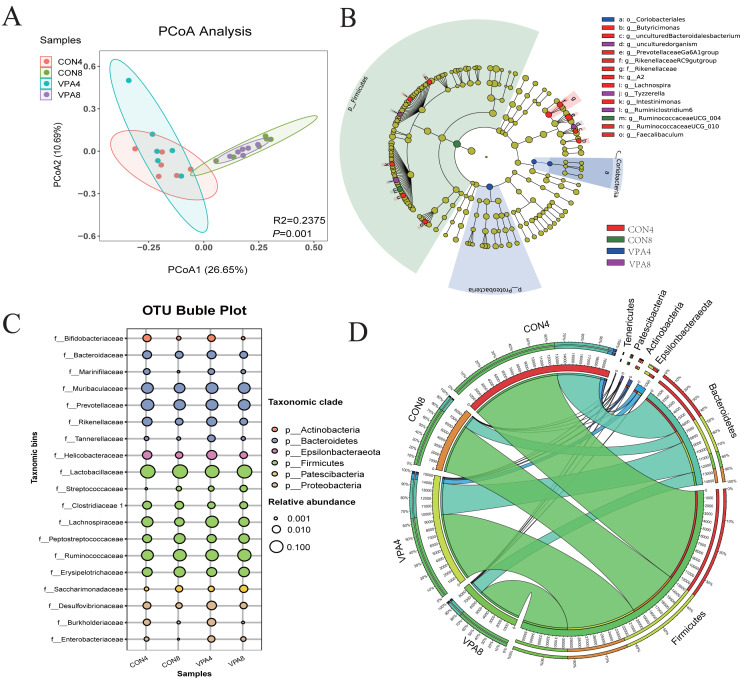
Microbiota analysis. (A) *β*-diversity was presented by PCoA. (B) Phylogenetic trees describing the taxonomic hierarchy of bacteria with significant differences between the two groups and logarithmic LDA scores of >2.00. (C) OTU Bubble chart of the gut microbiota at the phylum and genus levels. (D) Chord diagram: the abundance of dominant phyla.

The PCoA for *β*-diversity revealed the degree of individual differences in intestinal microflora structure at different ages in healthy and autistic-like groups. The PCoA results (Fig. 3A) revealed that the intestinal microbiota of rats lacked homogeneity in dispersion but still showed age-related clustering, with those of healthy and autistic-like rats of the same age clustered more closely together, indicating that the intestinal microflora composition of the two groups was similar at the same ages.

Figure 3B shows statistical differences in the biomarker species with LDA scores of >2 (the threshold for distinguishing a characteristic logarithm LDA score). Biomarker species with statistical differences changed in the feces of rats from four to eight weeks of age. The species with significant differences in the four-week-old healthy rats were those belonging to *Prevotellaceae Ga6A1 group*, *Rikenella*, *Rikenellaceae RC9 gut group*, *Butyricimonas*, *Lachnospira*, *Intestinimonas, Ruminococcaceae UCG-010*, and *Faecalibaculum*. Beyond this, the only biomarker species with statistical differences in the eight-week-old healthy rats were those belonging to *Ruminococcaceae UCG-004.* The species with significant differences in the four-week-old autistic-like rats were those belonging to *Coriobacteria coriobacteriales* and *Proteobacteria*, and in the eight-week-old autistic-like rats, those belonging to *Tyzzerella* and *Ruminiclostridium 6*.

**Figure 4:**
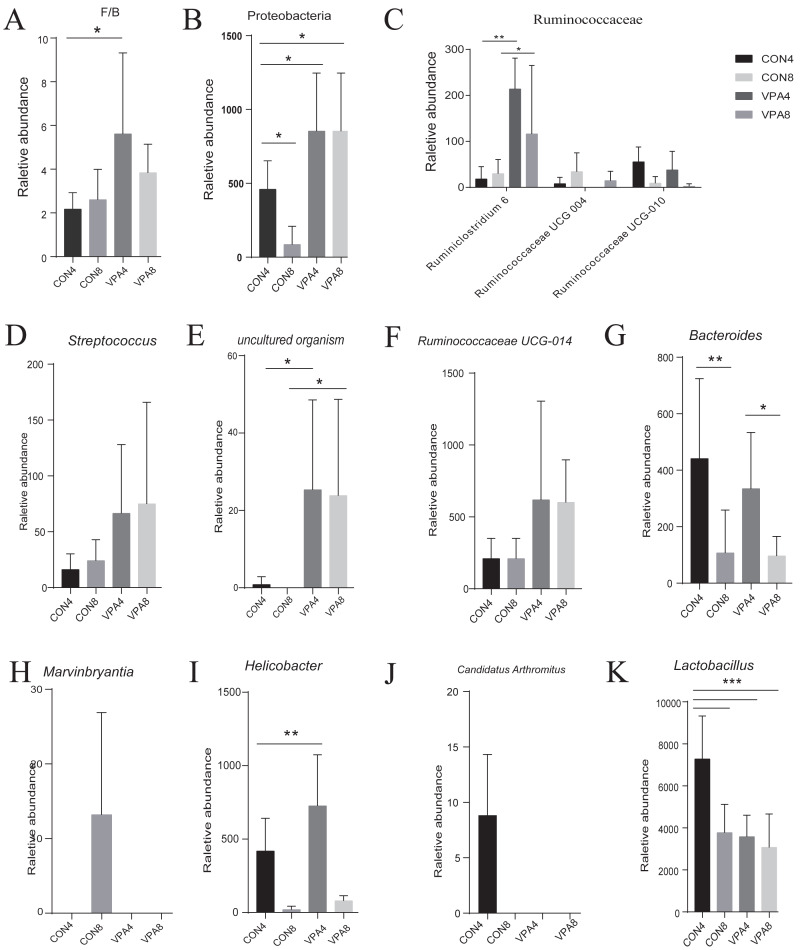
Microbiota analysis. (A) Firmicutes/Bacteroidetes ratio. (B–L) The abundances of characteristic gut microbiota from weaning to sexual maturation. Data represent mean with 95% CI. One-way ANOVA followed by Fisher’s LSD multiple-comparison test: *(*p* < 0.05); **(*p* < 0.01); ***(*p* < 0.001); ****(*p* < 0.0001).

At the phylum level (Figs. 3C and 3D), the abundances of both *Firmicutes* and *Bacteroidetes* showed a decreasing trend with age, and the F/B ratio in autistic-like group was higher than that in healthy group (Fig. 4A). The abundance of *Proteobacteria* showed a decreasing trend with age in healthy group but remained high in autistic-like group (Fig. 4B).

At the family level (Fig. 2B), the abundance of *Lactobacillaceae* was significantly lower in autistic-like group than in healthy group, whereas *Helicobacteraceae*, *Ruminococcaceae*, and *Lachnospiraceae* were more abundant in autistic-like group. The difference in the abundance of *Muribaculaceae* between autistic-like group and healthy group was not significant. In four weeks of age, *Prevotellaceae* was more abundant in autistic-like group than in healthy group, but the difference was not significant compared with that in eight weeks of age. Moreover, the abundance of *Prevotellaceae* in the autistic-like group did not vary significantly from four to eight weeks of age. From the fourth to eighth week, *Erysipelotrichaceae* was significantly less abundant in the autistic-like group than in healthy group, and *Enterobacteriaceae* was significantly more abundant in autistic-like group than in healthy group. Also, in the fourth week, *Streptococcaceae* and *Desulfovibrionaceae* were significantly more abundant in autistic-like group than in healthy group, but the abundance of *Desulfovibrionaceae* showed no significant difference between the two groups in the eighth week.

**Figure 5:**
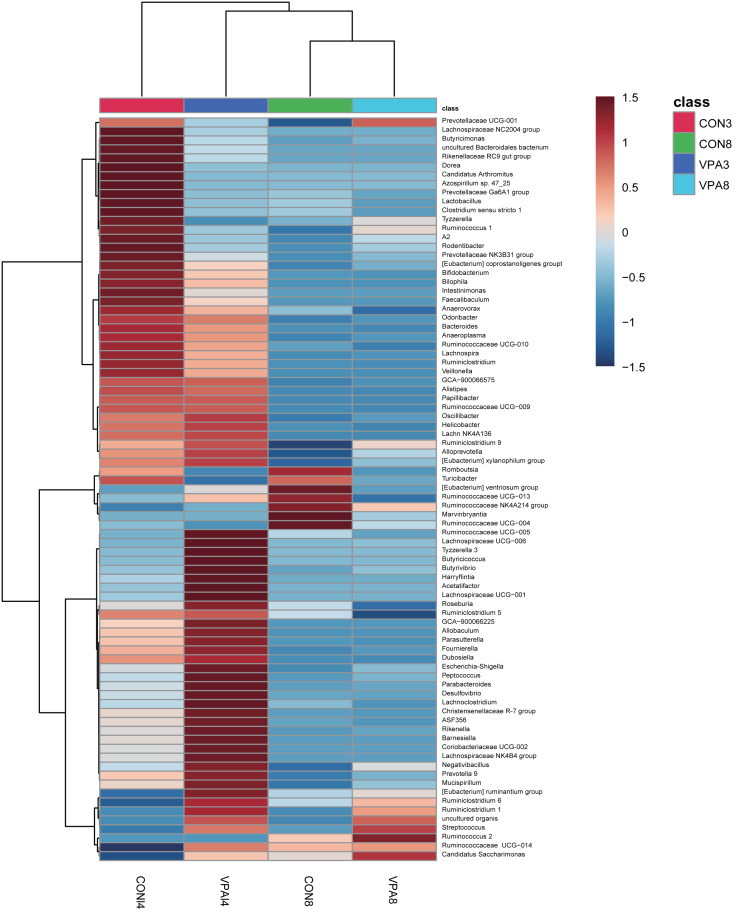
Heat map of the gut microbiota at the genus level of 4 and 8-week-old autistic rats and healthy rats. The colors represent the normalized relative abundance, with red being higher level and blue lower level. The clustering of group was made for investigate the affinity difference between groups at the level of genus.

At the genus level, the heat map in Fig. 5 shows there were significant differences in gut microbial abundance between the two groups at different ages, especially in four-week-old groups. The abundances of *Ruminiclostridium 6, Ruminococcaceae UCG-004, Streptococcus*, and *uncultured organism* belonging to the families *Muribaculaceae* and *Marvinbryantia* were different between autistic-like group and healthy group. In the fourth week, although Ruminococcaceae was more abundant in autistic group than healthy group, *Ruminiclostridium 6*, *Ruminococcaceae UCG-010* and *Ruminococcaceae UCG-004* were less abundant in autistic-like group (Fig. 4C). *Ruminococcaceae UCG-004* did not appear in autistic-like group until the eighth week, and even in the eighth week, its abundance was significantly lower than healthy group (Fig. 4C). *Streptococcus*, *uncultured organism*, and *Ruminococcaceae UCG-004* were more abundant (Figs. 4D, 4E and 4F) and *Bacteroides* was less abundant in autistic-like group than healthy group, especially after weaning (Fig. 4G). In the eighth week, *Marvinbryantia* and *Helicobacter* were less abundant in autistic group than healthy group (Figs. 4H and 4I). In the fourth week, *Candidatus Arthromitus* was presented only in healthy group and absent in autistic group (Fig. 4J). Meanwhile, in the same week, *Lactobacillus* was more abundant in healthy group than autistic-like group, but there was no difference in abundance between the eight-week-old healthy group and autistic group (Fig. 4K).

**Figure 6:**
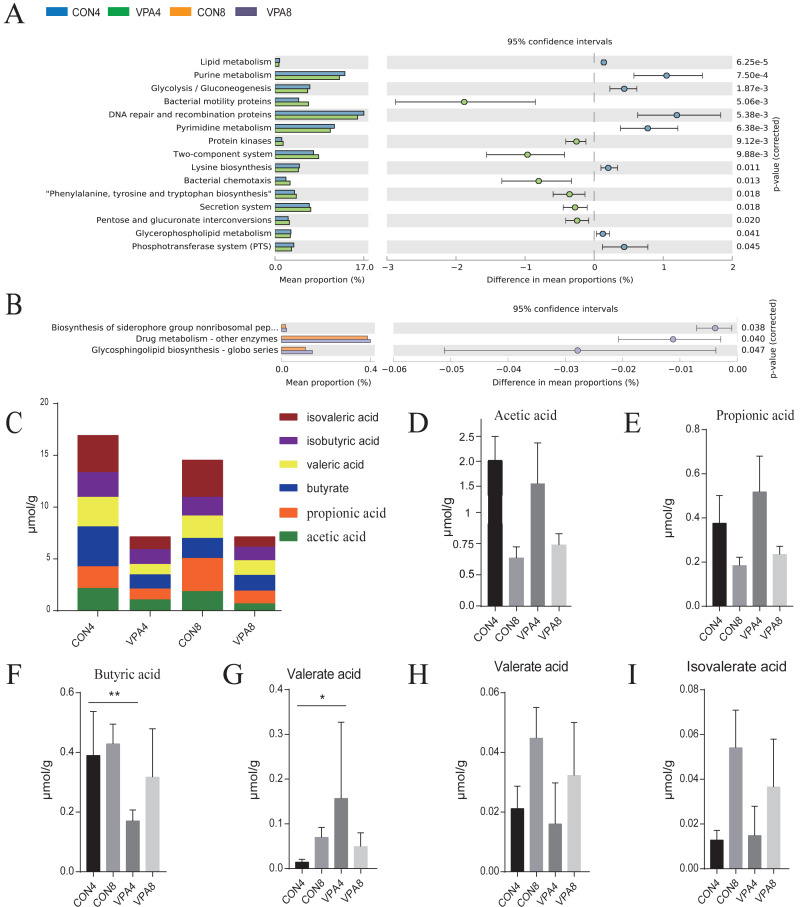
The prediction of metagenome function by PICRUST and SCFA analysis. (A) Differential KEGG pathways between 4- and 8-week-old rats were evaluated using a two-sided Welch’s *t*-test at *p* < 0.05. (B) Heat map of SCFAs. (C–H) The levels of acetic acid, propionic acid, butyric acid, valeric acid, isobutyric acid and isovaleric acid from weaning to sexual maturation. Data represent mean with 95% CI. One-way ANOVA followed by Fisher’s LSD multiple-comparison test: *(*p* < 0.05); **(*p* < 0.01); ***(*p* < 0.001); ****(*p* < 0.0001).

Functional prediction (Figs. 6A and 6B) revealed that, in the eighth week, the biosynthesis levels of siderophores, nonribosomal peptides, drug metabolism, and other enzymes, and *H*. *pylori* infection was lower in healthy group than in autistic group (*P* < 0.05). In the fourth week, lipid metabolism, purine metabolism, glycolysis, gluconeogenesis, and pyrimidine metabolism were significantly higher in healthy group than autistic group (*p* <0.0001).

### The butyric acid and propionic acid metabolisms were impaired from weaning to sexual maturation

The number and content of SCFAs depend mainly on the composition of gut microbiota. Compared with the healthy group, the autism group exhibited a decreased total amount of SCFAs in the feces from four to eight weeks of age (Figs. 6C and 6I). In the fourth week, the fecal acetic acid level was lower, whereas the fecal valeric acid, propionic acid, and isovalerate levels were higher in autistic group. The fecal butyric acid level was also significantly lower in autistic-like group. In the eighth week, the fecal propionic acid, isopentric acid, and acetic acid levels were higher, whereas the fecal butyric acid and isobutyric acid levels were lower in autistic-like group. Although the total butyric acid metabolism increased with sexual maturity in autistic-like group, the overall butyric acid level remained lower than that of healthy group, suggesting significant impairment in butyric acid metabolism. Besides, the fecal propionic acid level remained high in autistic group from weaning through sexual maturation.

### Helicobacter was a major biomarker from weaning to sexual maturation in autistic rats

**Figure 7:**
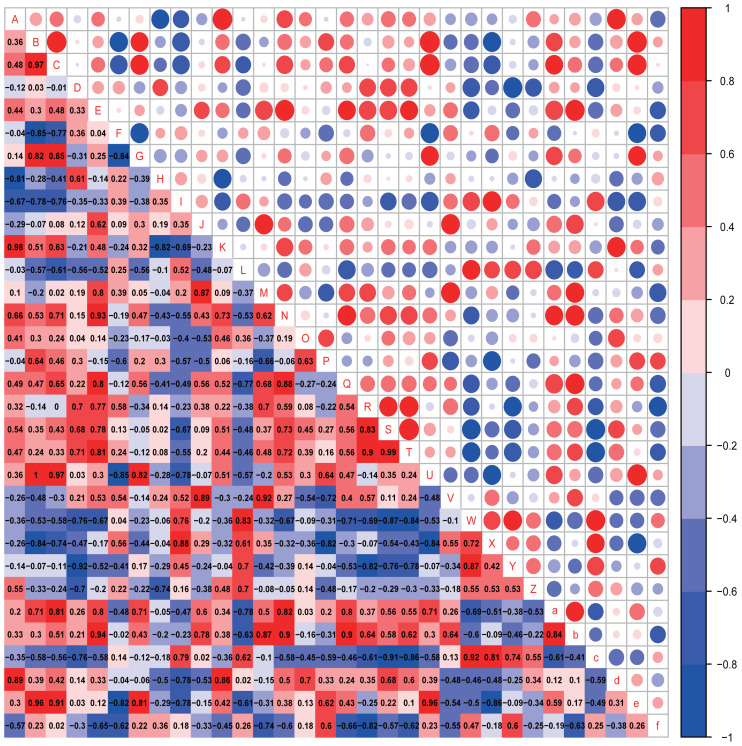
Correlation analysis. Correlation heatmap. Pearson correlation coefficients among behaviours, OTU abundance and function of 4-week-old autistic rats were present in Heatmap. The colors represent the correlation, with red being more positive and blue more negative. Significance is given as *(*p* < 0.05) and **(*p* < 0.01). In open-field test, the time spent in the middle are of an open field is measured to evaluate exploration behavior; the social index was the ratio of the residence time in the right side of wire cage to the residence time in the left cage; the time spend on the side of the novel object was used to assess cognitive ability; the number of times self-grooming is applied to assess repetitive or stereotyped behaviour; the number of times autistic rats defecated is a reflect of anxiety-like behaviour. (A: *Prevotellaceae UCG-001*; B: *Rikenellaceae RC9 gut group*; C: *Parabacteroides*; D: *Helicobacter*; E: *GCA-900066575*; F: *Lachnospiraceae NK4A136 group*; G: *[Eubacterium] ruminantium group*; H: *Fournierella*; I: *Harryflintia*; J: *Intestinimonas*; K:* Negativibacillus*; L: *Ruminiclostridium*; M: *Ruminococcaceae UCG-005*; N:*Ruminococcaceae UCG-014*; O: *[Eubacterium] coprostanoligenes group*; P: *Allobaculum*; Q: *Veillonella*; R: Epithelial cell signaling in *Helicobacter pylori* infection; S: Lipopolysaccharide biosynthesis; T: Lipopolysaccharide biosynthesis proteins; U: *Rikenellaceae RC9 gut group*; V: Grooming frequency; W: Exploration behaviour; X: Social index; Y: Novel object recognition behaviour; Z: Anxiety-like behaviour; a: Acetic acid; b: Propionic acid; c: Butyrate acid; d: Valeric acid; e: Isobutyric acid; f: Isovaleric acid).

**Figure 8:**
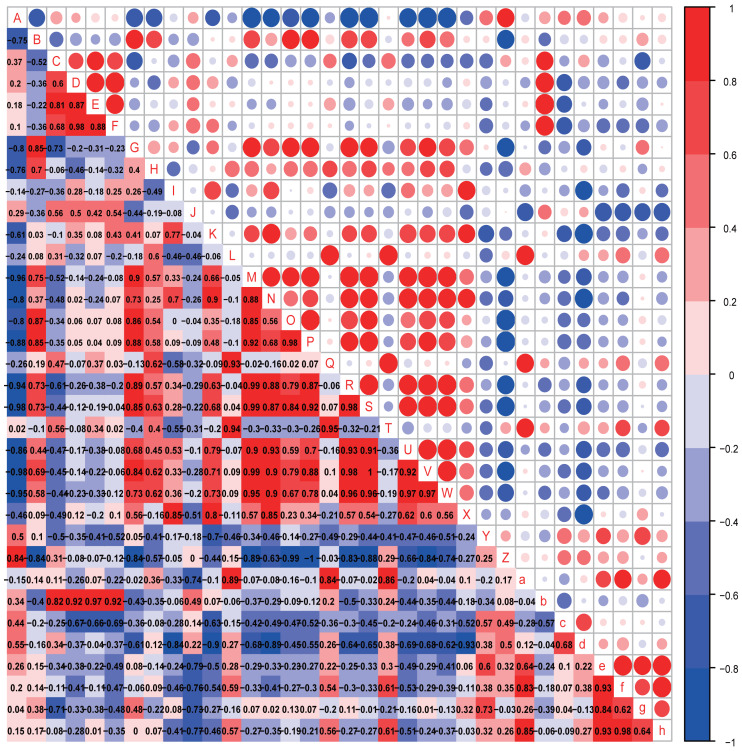
Correlation analysis. Correlation heatmap. Pearson correlation coefficients among behaviours, OTU abundance and function of 8-week-old autistic rats were present in Heatmap. The colors represent the correlation, with red being more positive and blue more negative. Significance is given as *(*p* < 0.05) and **(*p* < 0.01). In open-field test, the time spent in the middle are of an open field is measured to evaluate exploration behavior; the social index was the ratio of the residence time in the right side of wire cage to the residence time in the left cage; the time spend on the side of the novel object was used to assess cognitive ability; the number of times self-grooming is applied to assess repetitive or stereotyped behaviour; the number of times autistic rats defecated is a reflect of anxiety-like behaviour. (A: *Odoribacter*; B: *Prevotella 9*; C: *Prevotellaceae Ga6A1 group*; D: *Alistipes*; E: *Gemella*; F: *Roseburia*; G: *Eubacterium ruminantium group*; H: *Eubacterium coprostanoligenes group*; I: *Desulfovibrio*; J: *Helicobacter*; K: *Ruminococcaceae NK4A214 group*; L: Carotenoid biosynthesis; M: Epithelial cell signaling in *Helicobacter pylori* infection; N: Inositol phosphate metabolism; O: Lipopolysaccharide biosynthesis; P: Lipopolysaccharide biosynthesis proteins; Q: Nucleotide metabolism; R: Phenylalanine metabolism; S: Phenylalanine tyrosine and tryptophan biosynthesis; T: Phosphotransferase system PTS; U: Tryptophan metabolism; V: Valine, leucine and isoleucine biosynthesis;W: Valine, leucine and isoleucine degradation; X: Grooming frequency; Y: Exploration behaviour; Z: Social index; a: Novel object recognition behaviour; b: Anxiety like behaviour; c: Acetic acid; d: Propionic acid; e: Butyrate acid; f: Valeric acid; g: Isobutyric acid; h: Isovaleric acid).

The possible associations between the gut microbiota and autistic-like behaviors, including exploration behavior, repetitive stereotyped behavior, social behavior, novel object recognition behavior, and anxiety-like behavior, were analyzed (Figs. 7 and 8).

In the four-week-old autistic-like rats, repetitive or stereotyped behavior was significantly and positively correlated with the abundances of *Intestinimonas* (*r* = 0.875, *p* = 0.022) and *Ruminococcaceae UCG-005* (*r* = 0.820, *p* = 0.046); exploration behavior was positively correlated with the abundance of *Ruminiclostridium* (*r* = 0.829, *p* = 0.042); novel object recognition behavior (*r* = −0.829, *p* = 0.042) and anxiety-like behavior (*r* = −0.802, *p* = 0.050) were significantly and negatively correlated with the abundance of *Helicobacter*. Besides, we found that some presumptive functions of the gut microbial communities were associated with the behaviors in autistic-like group. The novel object recognition behavior was significantly negatively correlated with epithelial cell signaling in *H. pylori* infection. Exploration behavior was remarkably and negatively correlated with lipopolysaccharide (LPS) biosynthesis (*r* = −0.829, *p* = 0.042) and LPS biosynthesis proteins (*r* = −0.829, *p* = 0.042). Besides, the abundance of *Helicobacter* was positively correlated with LPS biosynthesis and LPS biosynthesis proteins. These findings suggest that *Helicobacter* abundance might function as an important biomarker involved in the regulation of LPS levels in the gut during the early progression of autism.

In the eight-week-old autistic-like rats, repetitive or stereotyped behavior was significantly and negatively correlated with the abundance of *Desulfovibrio* (*r* = 0.939, *p* = 0.005); social behavior was negatively and markedly correlated with the abundance of *[Eubacterium] ruminantium group* (*r* = −0.828, *p* = 0.042); anxiety-like behavior was positively correlated with the abundances of *Prevotellaceae Ga6A1 group* (*r* = 0.853, *p* = 0.031), *Alistipes* (*r* = 0.925, *p* = 0.008), *Gemella* (*r* = 0.870, *p* = 0.024) and *Roseburia* (*r* = 0.907, *p* = 0.012). Also, novel object recognition behaviors (*r* = −0.886, *p* = 0.019) were significantly correlated with the abundance of *Helicobacter*. This finding is consistent with that observed in the four-week-old autistic-like rats. Further, the abundance of *Ruminococcaceae NK4A214 group* was correlated with repetitive or stereotyped behavior (*r* = 0.772, *p* = 0.072). Novel object recognition behavior was positively correlated with carotenoid biosynthesis, nucleotide metabolism, and PTS. The following presumptive functions were negatively correlated with social behavior and positively with repetitive stereotyped behavior: alanine, aspartate and glutamate metabolism, epithelial cell signaling in *H*. *pylori* infection, LPS biosynthesis, LPS biosynthesis proteins, phenylalanine metabolism, phenylalanine, tyrosine and tryptophan biosynthesis, tryptophan metabolism, valine, leucine, and isoleucine biosynthesis, and valine, leucine, and isoleucine degradation. The disturbance in amino acid metabolism and LPS levels might play a vital role in autistic-like behaviors development.

Notably, the abundances of critical biomarkers found in four-week-old autistic-like rats, including *Intestinimonas*, *Ruminococcaceae UCG-005*, *Harryflintia*, *Ruminiclostridium*, *Rikenellaceae RC9 gut group*, and *GCA-900066575*, were almost negligible in the eight-week-old autistic-like rats. Besides, the correlations between autistic-like behaviors and the abundances of *Odoribacter*, *uncultured organism*, *Prevotellaceae Ga6A1 group*, *Alistipes*, and *Gemella* were inconsistent between the four- and eight-week-old groups. The changes in these biomarkers might be due to the differences in age and diet (breast milk vs. food). The diet of the offspring after weaning had been recently changed from breast milk to rat feed, and during this time, the structure and composition of gut microbiota were expected to be relatively unstable. Nonetheless, the association of some biomarkers with the behavioral stability in autistic-like rats remained throughout weaning and sexual maturation. In particular, the correlations between autistic-like behaviors and the abundances of *Helicobacter*, *Desulfovibrio*, *Prevotella 9*, *[Eubacterium] ruminantium group*, and *Roseburia* did not vary with diet and age (remained consistent from weaning to sexual maturation) and can thus be considered vital biomarkers for all age groups regardless of diet. Besides, inositol phosphate metabolism, amino acid metabolism, and LPS biosynthesis were the potential main metabolic pathways found to be associated with autistic-like behaviors.

### Unchanged relative levels of butyrate acid with diet or age compared with the healthy group during weaning and sexual maturation might drive autistic-like behaviors development

The possible associations between SCFA levels and autistic-like behaviors, including exploration behavior, repetitive stereotyped behavior, social behavior, novel object recognition behavior, and anxiety-like behavior, were analyzed (Figs. 7 and 8).

In the four-week-old autistic rats, the butyrate acid level was positively correlated with social behavior (*r* = 0.942, *p* = 0.005). This finding was consistent with the finding of a significant decrease in the butyric acid level in the feces of autistic-like group. Further, the acetic acid and propionic acid levels also showed a consistent correlation with exploration behavior, repetitive or stereotyped behavior, social behavior, and novel object recognition behavior, but the correlations were weak.

In the eight-week-old autistic-like rats, the acetic acid level was negatively correlated with repetitive or stereotyped behavior (*r* = −0.802, *p* = 0.054), and the valeric acid (*r* = 0.829, *p* = 0.042) and isovaleric acid (*r* = 0.943, *p* = 0.004) levels were positively correlated with novel object recognition behavior. The propionic acid levels showed consistent correlation with repetitive or stereotyped behavior (*r* = −0.926, *p* = 0.008).

These results indicate that the butyric acid level was consistently associated with autistic-like behaviors from weaning to maturation, whereas the acetic acid and propionic acid level showed opposite associations with autistic-like behaviors from weaning to sexual maturity. This result may be attributable to the changes during the adjustment period after weaning. The consistent low butyric acid level that remained from weaning through sexual maturation despite changes in age and diet suggests that butyric acid participates in autistic-like behaviors development.

## Discussion

In this study, the rat offspring exposed to 600 mg/kg VPA in utero developed malformed tails, delayed development, and autistic-like behaviors, starting with early repetitive stereotyped behavior. The malformation of tails was described as a ‘kinky tail’ (Foley et al., 2012), which helped confirm a successful exposure of VPA in the embryo (Schneider et al., 2008). In clinical studies, clicky hips, fixed flexion deformity of fingers and elbows, postural scoliosis and metatarsus primus varus have been observed in offspring whose mothers took >1 g of VPA per day during pregnancy (Kini et al., 2006). Notably, the microbial metabolites or other substances from the mother can transmit through the placenta and participate/interfere in the development of the fetus. When administered to the pregnant rats on E 12.5 in our study, VPA has been recognized as a potent teratogen that most notably induces neural tube defects in humans, mice, and other vertebrate embryos. And the loss of functions of the hippocampus, thalamus, frontal cortex, parietal cortex, etc. has been proved (Bittigau et al., 2002; Wang et al., 2011). The developmental characteristics and behaviors of these offspring were found to be similar to the clinical manifestations of autism.

Previous studies have reported gut microbiome in early life plays an important role in brain and behaviors development. Antibiotic interventions in mice from weaning to sexual maturation have been shown to change their brain chemistry and behaviors, and long-term antibiotic administrations could result in a decline in hippocampal neurogenesis and cognitive performance (Moehle et al., 2016). Correspondingly, germ-free mice have also been reported to show abnormal behaviors compared with normal mice (Luczynski et al., 2016). Therefore, the investigation of behavior-related gut microbial biomarkers is crucial. From the moment of delivery, the new-born is exposed to the microbes of the outside world. In the normal case, healthy development, gut microbiota composition of new-borns grows closer to that of their mother with age (Heijtz, 2016). However, when certain metabolites, such as VPA and LPS, interfere during development, the original microbial balance is disturbed, leading to behavioral deficits. Our results related to the abundance of the Bacteroidetes phylum were not consistent with those reported in previous studies, but the abundance of *Firmicutes* at eight weeks and the F/B ratio at two-time points were consistent with those in previous studies (Liu et al., 2019a; Liu et al., 2019b; Zhang et al., 2018). The study in Bacteroidetes phylum rather diverse and no clear consensus has been reached. The Bacteroidetes abundance in severe and mild autism children significantly increased compared to controls (Finegold et al., 2010). Some other studies observed either a decrease (Strati et al., 2017; Williams et al., 2011) or no significant differences (Gondalia et al., 2012; Son et al., 2015) between autism and controls in Bacteroidetes abundance. So more further research is needed to fully understand the impact of Bacteroidetes percentage in autism. Further, the differences in the abundances of *Ruminococcaceae*, *Lachnospiraceae*, and *Muribaculaceae* between the autism and healthy groups in our study were also not consistent with some results observed in autistic children (Zhang et al., 2018; Liu et al., 2019a; Liu et al., 2019b; Shindler et al., 2019). Although several studies have evaluated the intestinal dysbacteriosis of patients with ASD, no consensus has been reached on the specific composition of intestinal flora in these patients, which may vary due to age, diet, medication, geographical area, complications, and the severity of gastrointestinal symptoms in patients with nerve behavior and lack of consistency. Forty-four percent of autistic patients have symptoms of carbohydrate dyspepsia, which may be associated with the poor carbohydrate-degrading activities of intestinal microorganisms and enzymes (Precup & Vodnar, 2019). In this study, the abundance of *Bacteroides*, a genus involved in carbohydrate breakdown (Alfawaz et al., 2018), in autistic-like rats was low. And the Prevotellaceae showed a stable abundance in the intestines of the autistic-like rats from the fourth to eighth week in this study, and the *Prevotella* genus in this family is known to function symbiotically in carbohydrate metabolism and vitamin biosynthesis (Rios-Covian et al., 2017). Besides, *Candidatus Arthromitus* was present only in the four-week-old healthy rats. The presence and growth of *Candidatus Arthromitus* in the intestinal tract is related to the host’s (both animals and human infants) diurnal age. It has been reported that *Candidatus Arthromitus* can promote intestinal sIgA secretion and intestinal mucosal immune maturation (Fuentes et al., 2008). Our results showed no colonization of *Candidatus Arthromitus* in the four-week-old autistic rats, suggesting impairment in the early maturation of their immune system. This finding must be further confirmed in future experiments.

During development, the weaning stage is a crucial phase in which various factors, such as environment and diet, have major effects on the gut microbiota composition of offspring. The gut microbiota and bodily functions develop naturally with age. The diet of weaned rats changes from breast milk to fodder, causing a dynamic change in their gut microbiota to adapt to the environment. Our results showed significant differences in the gut microbiota composition between autistic-like rats and healthy rats during this phase, suggesting that autism may have a regulatory effect on the gut microbiota composition. We found that the gut microbiota composition and SCFA levels differ during weaning and sexual maturation between autistic-like and healthy rats. However, although some microbial biomarkers changed with age and diet, some remained consistent irrespective of age and diet. For example, from weaning to sexual maturation, novel object recognition behavior maintained a significant negative correlation with the abundance of *Helicobacter* and the presumptive functions of AD, PD, and Huntington’s disease. All of these neurodegenerative diseases, especially AD, are accompanied by a certain degree of cognitive impairment. Recent studies have reported *H. pylori* infection as a risk factor for AD (hazard ratio = 1.46, *p* = 0.040) and that treating the *H. pylori* infection could prolong the life of AD patients. Previous studies have confirmed that *H. pylori*: causes delayed maturation of dendritic spines in the hippocampus, promoting spatial learning and memory deficits in rats; interrupts synaptic function; and induces cognitive impairment by increasing the activity of *γ*-secretase and the level of A *β*_42_ in the hippocampus and cortex (Wang et al., 2014). Thus, the lower cognitive ability of autistic-like rats in the younger stage observed in our study may be attributable to the high abundance of *Helicobacter* during the periods of weaning and sexual maturation. This finding needs to be confirmed in further study.

Besides, we found that inositol phosphate metabolism was significantly positively associated with repetitive stereotyped behavior. The role of VPA in autism development involves the induction of neural tube defects, leading to nerve damage in the offspring. Studies have shown that neural tube defects are closely related to inositol metabolism and that abnormal inositol metabolism can activate the PI3K/AKT signaling pathway, mediate autophagy activity, and cause behavioral abnormalities (Zhang, Zhang & Zhang, 2016). Further, inositol 1,4,5-triphosphate (InsP3) from inositol phosphate metabolism is a vital secondary messenger in the InsP3/Ca2 signal transduction pathway (Shi et al., 2006). The disturbance of this pathway has been linked to bipolar affective disorder, AD, and PD. These findings suggest that the disruption of inositol phosphate metabolism is responsible for the repetitive or stereotyped behavior observed in autistic rats.

The *Odoribacter* genus plays a key role in social performance. The presumptive functions of the gut microbial communities are significantly related to the effects of *Odoribacter* on amino acid metabolism and LPS biosynthesis. Studies have reported that abnormal amino acid metabolism is closely related to neurological abnormalities (Gevi et al., 2016; Tărlungeanu et al., 2016). Regulation of amino acid levels has been shown to alleviate the behavioral disorders and neurological abnormalities of mice.

Tryptophan metabolism (Agus, Planchais & Sokol, 2018) involves three main pathways in the gastrointestinal tract: (1) tryptophan is directly converted into several metabolites by the gut microbiota, including ligands for aryl hydrocarbon receptors, which can help regulate the activity of glial cells; (2) indoleamine 2,3-dioxygenase 1, the L-tryptophan-degrading enzyme, mediates the immune and kynurenine pathway of epithelial cells; (3) tryptophan hydroxylase 1 present in enterochromaffin cells converts tryptophan to serotonin, a pathway that is usually disturbed in autism. Also, LPS biosynthesis can increase the level of inflammation in the host and interfere with nervous system development. Thus, amino acid metabolism and LPS biosynthesis might be the major factors involved in the development of social dysfunction with age in autism.

SCFAs, as microbial metabolites, can directly or indirectly participate in the brain-gut microbiota communication axis and have a major impact on the immune and endocrine systems. A recent study found that a mixture including sodium acetate, sodium propionate, and sodium butyrate could inhibit nerve inflammation and neuronal loss caused by a high-fructose diet in C57BL/6N mice (Li et al., 2019). Also, sodium butyrate could promote the transcription of inhibitory pathway transcripts to improve impaired social interactions of autistic BTBR mice (Kratsman, Getselter & Elliott, 2016). In this study, the levels of acetic acid, propionic acid, and isovaleric acid were not consistent at four and eight weeks of age, which might be related to the diet changes after weaning. However, in the autism group, the level of butyric acid was lower and the level of propionic acid was higher compared with the healthy group throughout this period, irrespective of variations in diet or age; thus, these acids might be involved in autism development. A study by De Theije et al. (2014) found that the level of butyric acid in the cecum was higher in an autistic BALB/C mouse induced by VPA on a gestational day 11. This is not consistent with our results, possibly due to the difference in pathological degree caused by the different modeling times or the different sampling areas, animal species, feeding conditions, and other factors. However, the investigation in the population shows that the butyric acid content of autistic patients is significantly down-regulated (Wang et al., 2020). Besides, we noted a significant correlation between the levels of SCFAs (butyrate acid, acetic acid, isobutyric acid, valeric acid, and isovaleric acid) and autistic-like behaviors in VPA-treated group, indicating that regulating the composition of SCFA-producing gut microbiota or directly supplementing the corresponding proportion of SCFAs might be a potential strategy to alleviate the neurological damage and behavioral abnormalities in autism.

## Conclusions

Neurological diseases are often associated with intestinal imbalances. We investigated the changes in the gut microbiota of autistic-like rats from weaning to sexual maturation. We found that gut microbiota and SCFA levels are closely related to each autistic-like behavior. The gut microbiota and SCFAs in autistic-like rats were different from those in healthy rats across all ages evaluated, and the characteristic microbiota and SCFAs related to autism varied with age. Although changes in diet might be the main factor for these variations during the first week after weaning, we found some microbiota and SCFAs did not change irrespective of variations in age and diet. These microbes and SCFAs could serve as biomarkers for autism in all age groups, regardless of diet. Beyond this, inositol phosphate metabolism, amino acid metabolism, and lipopolysaccharide biosynthesis may play a vital role for VPA-induced autism. This knowledge will have a positive impact on further explorations of treatment models of the intestinal environment for autistic patients.

##  Supplemental Information

10.7717/peerj.11103/supp-1 Supplemental Information 1 Metadata map for gut microbiota analysis

10.7717/peerj.11103/supp-2 Data S1 Raw data for fertility rate, weight, behaviors and the level of SCFAs

10.7717/peerj.11103/supp-3 Supplemental Information 3 P-values for Figure 7

10.7717/peerj.11103/supp-4 Supplemental Information 4 P-values for Figure 8

10.7717/peerj.11103/supp-5 Figure S1 Overview of the experimental procedures

## Additional Information and Declarations

### Data Availability

The following information was supplied regarding data availability: Data is available at the National Center for Biotechnology Information (NCBI): PRJNA606860, SRR11277867. Raw data is available in the Supplemental Files.
