# Supplementary figures and images for: The autistic-like behaviors development during weaning and sexual maturation in VPA-induced autistic-like rats is accompanied by gut microbiota dysbiosis
Source: PeerJ. 2021 May 3;9:e11103. doi: 10.7717/peerj.11103 (PMC8101471; doi:10.7717/peerj.11103)

- Body weight monitoring (P 7d, 21d, 56d)
- Incline test (P 7d, 8d, 9d, 10d)
- Swimming coordination (P 7d, 11d, 13d)

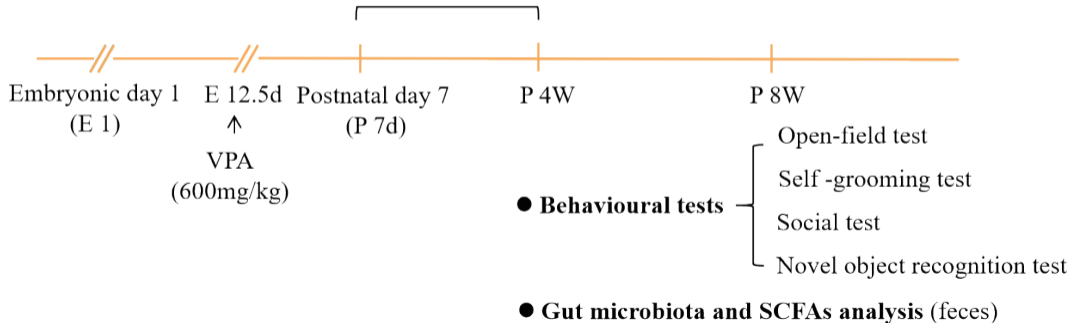

Supplement: Figure S1 [file peerj-09-11103-s005.pdf]
